# Deaths from Violence

**Published:** 1913-10

**Authors:** 


### Deaths From Violence

"Physician, heal thyself," is an irony older than the English language. At present, the admonition needed is to avoid death by violence. For the entire population, violent deaths comprise about 7.5 per cent. of the total. Of these, about one in six is by suicide. Theoretically, physicians should have a mortality by violence corresponding to the more fortunate class of the population. Actually, their mortality by suicide alone is almost as high as that of the entire population by all forms of violence. Judging from recent reports—it is too early to present statistics—automobile and other accidents and deliberate killing are removing altogether too large a number of our profession. Are we wrong, then, in suggesting greater caution, greater self control, perhaps even safer associations?

